# Anticancer drug therapy for anaplastic thyroid cancer

**DOI:** 10.1530/ETJ-24-0287

**Published:** 2025-04-14

**Authors:** Naomi Kiyota, Taiji Koyama, Iwao Sugitani

**Affiliations:** ^1^ Department of Medical Oncology/Hematology, Kobe University Hospital, Kobe, Japan; ^2^ Kobe University Hospital Cancer Center, Kobe, Japan; ^3^ Department of Endocrine Surgery, Nippon Medical School, Tokyo, Japan

**Keywords:** anaplastic thyroid cancer, anticancer treatment, chemotherapy, molecular targeted agent, *BRAF* V600E, immune checkpoint inhibitor

## Abstract

Anaplastic thyroid cancer is one of the rarest subtypes of thyroid cancer, accounting for only 1–2% of all thyroid cancer cases. It is also one of the most aggressive: prognosis remains dismal and the disease-specific mortality rate is close to 100%. This rarity has markedly limited the availability of prospective trial results, and no standard chemotherapeutic option for unresectable or metastatic anaplastic thyroid cancer has yet been established. Nevertheless, combination therapy with a BRAF inhibitor and MEK inhibitor has shown encouraging efficacy in patients with *BRAF* V600E-mutated anaplastic thyroid cancer. Other novel treatments such as immune checkpoint inhibitors have also shown promising results. Owing to these therapeutic advances, the prognosis of anaplastic thyroid cancer appears to be gradually improving. However, further development of novel treatments for this rare malignancy requires the development of substantial infrastructure for international collaborative study.

## Introduction

Anaplastic thyroid cancer (ATC) is one of the rarest and most aggressive subtypes of thyroid cancer (TC) (1). According to the staging guidelines of the American Joint Committee of Cancer (AJCC), all patients diagnosed with ATC are classified as having stage IV disease (AJCC 8th) (2). Although ATC accounts for only 1–2% of TC (3), historic disease-specific mortality is nearly 100% (1, 4, 5, 6), making it the most frequent cause of death among all TC patients (7). Indeed, a retrospective study using a database of the French ENDOCAN-TUTHYREF network (*n* = 360) demonstrated a median overall survival (OS) for ATC patients of 6.8 months. Furthermore, in patients with a high neutrophil–lymphocyte ratio – known as a poor prognostic inflammatory marker of peripheral blood, which might reflect the tumor immune microenvironment (TIME) – median OS with stage IVB/IVC disease was only 2.9 months (8, 9). If permitted by the patient’s condition, multimodal therapy including surgery, radiation therapy and chemotherapeutic agents plays a critical role in the management of ATC and may improve treatment outcomes for patients (10). Indeed, neoadjuvant approach with dabrafenib plus trametinib and pembrolizumab showed promising treatment outcomes with a median OS of 63 months and 2-year survival of 74.5% (11). However, this kind of aggressive multimodal approach may also carry the risk of adversely affecting the patient’s quality of life, and a shared decision-making approach with patients is therefore essential. Furthermore, palliative chemotherapy has an important role for patients with ATC. However, very few randomized-controlled trials (RCTs) for this extremely rare malignancy have been conducted, and no standard chemotherapeutic option for unresectable or metastatic ATC based on RCTs has accordingly been established. However, combination therapy with a BRAF inhibitor and MEK inhibitor has shown encouraging efficacy for *BRAF* V600E-mutated ATC (12, 13, 14), which accounts for around 40–60% of ATC (15, 16, 17), and this combination has become a standard treatment option for *BRAF* V600E-mutated ATC. Furthermore, other novel treatment options such as immune checkpoint inhibitors (ICIs) have also shown promising results (11, 18, 19). Thanks to recent advances in these novel therapeutic options, the prognosis of ATC patients appears to be improving year by year (1). Here, this review focuses on current evidence and future perspectives of anticancer drug therapy for ATC.

## Anticancer treatment options for ATC

### Cytotoxic agents

Although several prospective clinical trials for patients with ATC have been conducted, the efficacy of cytotoxic chemotherapy for ATC is limited (Table 1). Agents investigated to date include cytotoxic agents such as doxorubicin, taxanes and their combination with platinum agents (20, 21, 22, 23, 24, 25). Although most of these agents showed only a modest response of short duration, paclitaxel and doxorubicin are possible treatment options for ATC patients without driver gene alterations such as *BRAF* V600E, *NTRK* fusion and *RET* fusion (12, 13, 14, 26, 27).

**Table 1:** Prospective studies of cytotoxic chemotherapy for ATC (20, 21, 22, 24, 25).

Treatment regimen	*n*	Response rate	PFS
Doxorubicin	41	17%	-
Doxorubicin + cisplatin	43	26%	-
Docetaxel	7	14%	6 weeks
Weekly paclitaxel	56	21%	1.6 months
Paclitaxel 96h	19	53%	-
Paclitaxel + carboplatin	25	16%	3.1 months
Paclitaxel + carboplatin + fosbretabulin	55	20%	3.2 months

*n*, number of patients; PFS, progression-free survival; ATC, anaplastic thyroid cancer.

### Molecular targeting agents

Vascular endothelial growth factor (VEGF) and its receptor (VEGF receptor; VEGF-R) play an important role in tumor growth and metastasis. VEGF-R is known to be overexpressed in ATC and VEGF-R inhibitors showed significant tumor growth inhibition in preclinical models (28). In clinical trials, several molecular targeting agents have been investigated for patients with ATC (Table 2) (29, 30, 31, 32, 33, 34). Among them, lenvatinib showed the most promising results, with a response rate of 24% in the initial report of a Japanese phase II trial. This lead to the approval of lenvatinib for ATC in Japan (32). However, subsequent phase II trials of lenvatinib for ATC from the United States and Japan showed disappointing results (33, 34). The reason why initial excitement over lenvatinib monotherapy diminished in subsequent clinical trials is uncertain. However, a French group reported that the differences in disease aggressiveness and response to lenvatinib might be related to the mixed pathology of ATC, including poorly differentiated or differentiated components (35). Another approach used VEGF-R inhibitors to enhance the efficacy of chemoradiotherapy for ATC. NRG/RTOG 0912 was a double-blinded randomized phase II trial (*n* = 71) to analyze the additional effects of pazopanib, a potent VEGF-R inhibitor, in chemoradiotherapy with paclitaxel. Although no significant additional effect of pazopanib was demonstrated, the treatment combination appeared to be feasible and safe (36).

**Table 2:** Molecular targeting agents for ATC (12, 13, 14, 26, 27, 29, 30, 31, 32, 33, 34, 52).

Treatment regimen	*n*	Response rate	MST	Note
Imatinib	11	25%	45% (6 months)	
Pazopanib	15	0%	3.7 months	
Sorafenib	20	10%	3.9 months	
Lenvatinib	17	24%	10.6 months	
Lenvatinib	28	3%	3.2 months	
Lenvatinib	42	12%	12% (12 months)	
Dabrafenib/trametinib	36	56%	14.5 months	*BRAF* V600E+
Encorafenib/binimetinib				
ATC	5	80% (4/5)	NR	*BRAF* V600E+
Total	22			
Vemurafenib/cobimetinib + atezolizumab				
Cohort 1	19	50%	43.2 months	*BRAF* V600E+
Total	43			
Larotrectinib				
ATC	7	29% (2/7)	NE	*NTRK* fusion+
Total	29			
Selpercatinib				
ATC	2	50% (1/2)	NE	*RET* fusion+
Total	19			

*n*, number of patients; MST, median survival time; NR, not reached; NE, not evaluable; ATC, anaplastic thyroid cancer.

With regard to the genomic landscape of ATC, previous reports have frequently described genomic alterations in *TP53*, *TERT*, *BRAF*, *NRAS* and *PIK3CA* among others (17, 37). Of these, *BRAF* V600E is a well-known driver gene alteration, which is found in 40–60% of ATC patients (15, 16, 17). In the phase II ROAR basket study, dabrafenib plus trametinib, namely combination therapy with a BRAF inhibitor and MEK inhibitor, showed very encouraging efficacy, with an overall response rate (ORR) of 56%, but with a relatively short median progression-free survival of 6.7 months and median OS of 14.5 months for patients with unresectable or metastatic *BRAF* V600E-mutated ATC (*n* = 36) (12). Furthermore, a Japanese phase II trial of encorafenib plus binimetinib, another combination therapy with a BRAF inhibitor and MEK inhibitor, showed promising efficacy, with an ORR of 55% (differentiated thyroid cancer (DTC) 47% (*n* = 17), ATC 80% (*n* = 5)) (14). These reproducible results support the further use of BRAF/MEK inhibitors for *BRAF* V600E-mutated ATC. The FDA (Food and Drug Administration) approved dabrafenib plus trametinib not only for *BRAF* V600E-mutated ATC patients but also for *BRAF* V600E-mutated DTC patients with no satisfactory treatment options. In addition, the Japanese PMDA (Pharmaceuticals and Medical Devices Agency) approved encorafenib plus binimetinib not only for *BRAF* V600E-mutated ATC patients but also for *BRAF* V600E-mutated DTC patients who were refractory to previous treatment options. Although the incidence of *NTRK* fusion and *RET* fusion in ATC is very low, ATC patients harboring *NTRK* fusion or *RET* fusion showed a response to a TRK inhibitor or RET inhibitor (26, 27). Since *BRAF* V600E mutation is the most prevalent gene alteration in ATC, immediate detection of *BRAF* V600E mutation is crucial for the management of ATC. Immunohistochemistry (IHC) of *BRAF* V600E is reliable, fast and cheap. A meta-analysis reported a pooled sensitivity of 96.8% and pooled specificity of 86.3%. A negative test largely excludes the mutation (38), and IHC test is useful for confirmation in clinical practice. Another useful non-tissue-based gene testing is liquid biopsy, which collects circulating tumor DNA to detect mutations such as *BRAF*-V600E. Liquid biopsy is a faster method than tissue-based mutation assay. A report from the MD Anderson Cancer Center (MDACC) showed that BRAF V600E-mutated cell-free DNA was highly concordant with the mutation detected with tissue-based assay at over 90% (39). Therefore, immediate submission to genetic testing to explore driver gene alterations is essential to identifying treatment options for this extremely aggressive malignancy. In fact, all published guidelines recommend rapid evaluation of patients with ATC at a high-volume center with expertise in treating ATC because of its extremely aggressive nature (40, 41, 42). For example, the Facilitating ATC Treatment (FAST) program established at MDACC decreased the access time and the number of successful referrals for ATC increased. The team also recommended the rapid identification of *BRAF* V600E-mutated ATC and the timely initiation of treatment for ATC (43, 44).

### Immune checkpoint inhibitors

ICIs such as anti-programmed death-1 (PD-1) antibodies (Abs) and anti-programmed death ligand 1 (PD-L1) Abs have been approved for various malignancies. There have been a few clinical trial results of ICIs for patient with advanced TC. The phase Ib KEYNOTE-028 trial assessed the safety and efficacy of pembrolizumab for patients with PD-L1-positive advanced DTC. Pembrolizumab showed a modest response, with an ORR of 9% (45). Another phase II trial, KEYNOTE-158, reported the result in a DTC cohort (*n* = 103). In this trial, pembrolizumab also showed a modest response, with an ORR of 7%, and the response did not differ by PD-L1 positivity (ORR of PD-L1-positive 9%, PD-L1-negative 6%). However, the median duration of response was 18.4 months. Therefore, it is essential to find an optimal biomarker for ICIs (46). On the other hand, ATC appeared to be more responsive to ICIs than DTC. Indeed, spartalizumab, one of the anti-PD-1 Abs, was investigated for its efficacy and safety in a phase I/II trial in patients with unresectable or metastatic ATC. Spartalizumab showed encouraging results, with an ORR of 19% (PD-L1-positive; 29%, PD-L1-negative; 0%), irrespective of BRAF mutation status (19). One possible explanation for the better efficacy of anti-PD-1 Abs in ATC patients is that the TIME of ATC may be suitable for immune checkpoint inhibition. For example, CXCL13-positive T lymphocytes were enriched in ATC and might promote the development of early tertiary lymphoid structures in TIME, which is reported to play an important role in antitumor immune response (47, 48). A second possible explanation for the better efficacy of ICIs in ATC is that tumor mutational burden (TMB) is higher in ATC than in poorly differentiated thyroid cancer (PDTC), given that TMB is a biomarker for the efficacy of ICIs (37, 49). A third possible explanation for the better efficacy of ICIs in ATC is that PD-L1 expression in tumor cells, which is a well-known biomarker for ICIs, is reportedly positive in 65–81% of ATC patients (19, 50, 51). Another attractive therapeutic approach is the combination of anti-PD-1 Abs with molecular targeting agents. In a retrospective analysis from MD Anderson Cancer Center (MDACC), 12 patients who were refractory to tyrosine kinase inhibitors (TKIs: lenvatinib, dabrafenib plus trametinib and trametinib alone) received a combination of pembrolizumab plus TKIs. Among them, five patients showed an objective response to the combination of TKIs with pembrolizumab (18). Furthermore, another retrospective study from MDACC (*n* = 71), which compared the dabrafenib plus trametinib with pembrolizumab and dabrafenib plus trametinib for *BRAF* V600E-mutated ATC, reported that triple combination therapy showed the most promising efficacy, with an ORR of 73% and OS of 17 months. In total, 23 patients received surgical resection after the triple combination and 11 of 22 patients (50%) with available pathological results showed a pathological complete response (11). Another phase II trial by MDACC of vemurafenib plus cobimetinib with PD-L1 antibody of atezolizumab showed an ORR of 50% and OS of 43.2 months in a cohort of *BRAF* V600E-mutated ATC patients (*n* = 19) (52). A phase II trial of neoadjuvant pembrolizumab and dabrafenib plus trametinib for patients with *BRAF* V600E-mutated ATC is nearing completion (NCT04675710).

## Ongoing clinical trials for novel treatment strategy for ATC

As mentioned above, anti-PD-1 Abs plus molecular targeting agents are now being investigated (Table 3). In addition to combination with BRAF/MEK inhibitors, combination therapy of anti-PD-1/PD-L1 Abs with VEGF pathway inhibitors appears promising, given previous findings of a synergistic effect and significant improvement in treatment outcomes in randomized-clinical trials for renal cell carcinoma, hepatocellular carcinoma and endometrial carcinoma (53, 54, 55). Although the peer-reviewed results have yet to be published, a phase II trial of ATLEP investigating the combination of lenvatinib and pembrolizumab for anaplastic and PDTC was reported at European Society of Medical Oncology (ESMO) 2022, and the combination showed a promising efficacy, with an ORR of 52% for ATC and 75% for PDTC (56). Other phase II trials of lenvatinib plus anti-PD-1 Abs for unresectable or metastatic ATC are now underway (Table 3).

**Table 3:** Ongoing clinical trials for unresectable or metastatic ATC.

NCT number	Patient	Treatment	Phase	*n*	Primary endpoint
NCT06374602	ATC	Pembrolizumab + lenvatinib	II	20	ORR
NCT04171622	ATC	Pembrolizumab + lenvatinib	II	25	OS
NCT05696548	ATC	Nivolumab + lenvatinib	II	51	ORR
NCT05119296	ATC	Pembrolizumab	II	20	ORR
NCT04238624	BRAF-mutated ATC	Cemiplimab + dabrafenib/trametinib	II	15	ORR
NCT05102292	BRAF-mutated ATC	HLX208, a BRAF inhibitor	I/II	25	ORR
NCT03085056	ATC	Trametinib + paclitaxel	I	13	PFS
NCT04552769	ATC	Abemaciclib	II	17	ORR
NCT06235216					
Cohort A	DTC	Sacituzumab govitecan	II	21	ORR
Cohort B	ATC			21	
NCT06007924	RAIR-DTC, ATC	Avutometinib + defactinib	II	30	ORR
NCT04420754	PDTC and ATC	AIC100 CAR-T (ICAM-1-directed)	I	70	AEs
NCT03449108	OC, TNBC and ATC	LN-145 or LN-145-S1 (TIL)	II	80	ORR

DTC, differentiated thyroid cancer; RAIR, radioactive iodine refractory; PDTC, poorly differentiated thyroid cancer; OC, ovarian cancer; TNBC, triple negative breast cancer; TIL, tumor infiltrative lymphocyte; ORR, objective response rate; PFS, progression-free survival; OS, overall survival; AEs, adverse events; ATC, anaplastic thyroid cancer.

**Figure 1:**
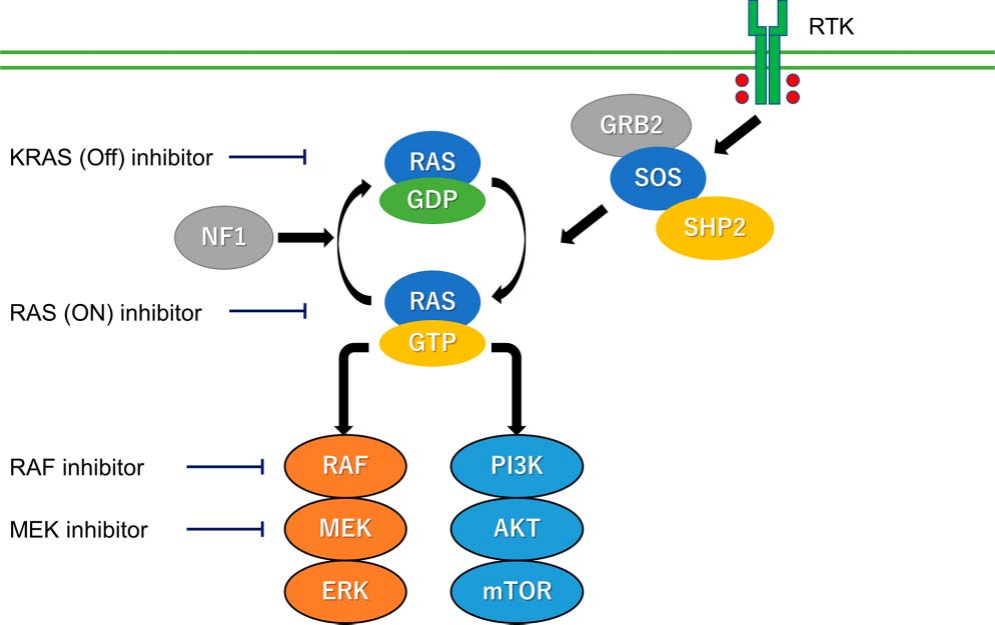
Signal pathway in *RAS*/*RAF*-mutated TC. RTK, receptor tyrosine kinase.

Thanks to the BRAF/MEK inhibitors, the prognosis of *BRAF* V600E-mutated ATC has improved. However, unmet needs remain for ATC patients with *BRAF* V600E mutation refractory to BRAF/MEK inhibitors and with *RAS* mutation. Regarding resistance against BRAF/MEK inhibition, pan-RAF inhibitors are now under extensive investigation. Currently approved BRAF inhibitors are potent against class I *BRAF* V600 mutants, which function as monomers. However, a possible mechanism of acquired resistance is new genetic alterations that induce BRAF dimerization. PF-07799933 is a novel pan-BRAF inhibitor which shows antitumor activity against *BRAF* V600- and non-V600-mutant cancers preclinically and in treatment refractory patients, including TC patients (57). *RAS* mutation is a frequent gene alteration in ATC and other cancer types, including lung cancer, colorectal cancer and pancreatic cancer. Its prevalence in ATC is around 20–25% and the frequency of the isoforms of *RAS* mutation are 14–18% in *NRAS*, 3–6% in *HRAS* and 4–6% in *KRAS* (17, 58). The prognosis of *RAS*-mutated ATC is extremely poor, and no effective therapeutic option for this population is currently available. Although *RAS*-activating mutation is identified as a primary driver of oncogenesis in many cancer types, *RAS* mutation has long been an undruggable target. However, sotorasib and adagrasib, which are *KRAS* G12C inhibitors, initially showed efficacy in non-small cell lung cancer with *KRAS* G12C mutation (59, 60). These clinically available *KRAS* G12C inhibitors are inactive GDP-bound mutant *KRAS* (so-called KRAS-off inhibitors) since the development of GTP-competitive RAS inhibitors were unfeasible due to high GTP-binding affinity (61). Furthermore, most of the affected tumors develop resistance to these KRAS-off inhibitors by the reactivation of RAS pathway. However, RAS-on inhibitors, which can inhibit all three active GTP-bound isoforms, are now under investigation in clinical trials and are expected to overcome RAS-related resistance mechanisms (Fig. 1). Table 4 shows ongoing clinical trials RAS/RAF-mutated solid tumors, including TC.

**Table 4:** Ongoing trials for RAS/RAF-mutated solid tumors, including TC.

NCT number	Treatment	Mode of action	Subject	Study phase
NCT05907304	Naporafenib plus trametinib	Pan RAF inhibitor plus MEK inhibitor	RAS Q61X-mutated solid tumor	1
NCT06270082	IK-595	Dual MEK/RAF inhibitor	RAS- or RAF-mutated solid tumor	1
NCT05585320	IMM-1-104	MEK1/2 inhibitor	RAS-mutated solid tumor	1/2
NCT06299839	PAS-004	MEK1/2 inhibitor	RAS/NF1/RAF-mutated solid tumor	1
NCT05379985	RMC-6236	Pan RAS (ON) inhibitor	KRAS G12X- and RAS-mutated solid tumor	1
NCT06096974	YL-17231	Pan RAS inhibitor	KRAS/HRAS/NRAS-mutated solid tumor	1/2

TC, thyroid cancer.

Antibody drug conjugates (ADCs) are composed of three key elements: a monoclonal antibody which binds to target antigen; a covalent linker to ensure that the payload is not prematurely released in the blood but rather within a tumor cell; and a cytotoxic payload that induces tumor cell apoptosis (62). ADCs have shown promise in other cancer types including breast cancer, gastric cancer and urothelial cancer (63, 64, 65, 66, 67, 68, 69, 70). Recently, ADCs have been under extensive development for many cancer types including TC (62). ADC is now an essential treatment strategy and combination with ICIs has shown a synergistic effect on immunogenic cell death and improved survival in urothelial carcinoma (62, 71). Regarding ATC, the TROP-2 (trophoblast cell-surface antigen 2)-directed ADC sacituzumab govitecan is now under investigation for patients with both previously treated DTC and ATC (NCT06235216). Further supporting the development of TROP-2-directed ADC in ATC, TROP-2 is expressed in 50–65% of ATC and TROP-2 overexpression is suggested to be associated with *BRAF* V600E mutation and aggressive behavior in papillary TC (72, 73, 74).

Adoptive cell therapies such as chimeric antigen receptor T cell therapy (CAR-T) utilizes engineered T cells to target specific antigens such as CD19 or B-cell maturation antigen (BCMA). This treatment has demonstrated high efficacy and gained approval for hematological malignancies (75, 76, 77). However, the application of adoptive cell therapy – including CAR-T therapy – to solid malignancies has not met the expectations raised in hematological malignancies and many challenges in the development of CAR-T therapy for solid malignancies remain. Nevertheless, CAR-T therapy is now under development for patients with DTC who are refractory to standard treatment and for patients with ATC. For example, ICAM-1 is a member of the immunoglobulin superfamily, which is known to play a role in the mediation of cell–cell interactions. ICAM-1 expression is highly correlated with adverse prognostic outcomes in TC patients. Furthermore, ICAM-1-directed CAR-T cells have shown significant therapeutic efficacy in animal models bearing ATC patient-specific tumors (78). Another approach to CAR-T therapy for TC is the targeting of TSH receptor. TSH receptor is highly expressed on papillary TC, and a preclinical study of TSH-R-targeted CAR-T therapy has shown therapeutic efficacy *in vivo* (79). AIC100 is an ICAM-1-directed CAR-T cell and a phase I trial of AIC100 for poorly differentiated TC and ATC is now underway (NCT05530754).

## Conclusion and future perspectives

ATC is one of the rarest and most aggressive malignancies and its prognosis remains dismal. Nevertheless, many clinical trials for ATC are ongoing and treatment outcomes are gradually improving year by year. Most clinical trials, to date, have been small, however, with fewer than 100 patients. Further efforts to develop novel treatments for this malignancy await the development of substantial infrastructure for international collaborative investigation.

## Declaration of interest

NK reports grants from Ono Pharmaceutical, Bristol-Meyers Squibb, Astra Zeneca, Chugai Pharmaceutical, Boehringer-Ingelheim, Bayer, GSK and Adlai Nortye outside the submitted work; and honoraria from Bayer, Ono Pharmaceutical, Bristol-Meyers Squibb, Novartis, Eisai, Merck Biopharma, Astra-Zeneca and Merck Sharp & Dohme. IS reports honoraria from Ono Pharmaceutical, Novartis and Eisai.

## Funding

This work did not receive any specific grant from any funding agency in the public, commercial or not-for-profit sector.

## Author contribution statement

NK made substantial contributions to the conception, drafting and reviewing of the manuscript. TK and IS made contributions to the drafting and reviewing of the manuscript.
